# Improved Models of Mini Anion Exchange Centrifugation Technique (mAECT) and Modified Single Centrifugation (MSC) for Sleeping Sickness Diagnosis and Staging

**DOI:** 10.1371/journal.pntd.0000471

**Published:** 2009-11-24

**Authors:** Philippe Büscher, Dieudonné Mumba Ngoyi, Jacques Kaboré, Veerle Lejon, Jo Robays, Vincent Jamonneau, Nicolas Bebronne, Wim Van der Veken, Sylvain Biéler

**Affiliations:** 1 Departments of Parasitology and Public Health, Institute of Tropical Medicine, Antwerp, Belgium; 2 Institut National de Recherche Biomédicale, Kinshasa, Democratic Republic of the Congo; 3 Institut de Recherche pour le Développement (IRD) UMR 177, Centre International de Recherche-Développement sur l'Elevage en zone Sub-humide (CIRDES), Bobo-Dioulasso, Burkina Faso; 4 Belgian Technical Cooperation, Kinshasa, Democratic Republic of the Congo; 5 Foundation for Innovative New Diagnostics, Geneva, Switzerland; New York University School of Medicine, United States of America

Human African trypanosomiasis (HAT), or sleeping sickness, is caused by two subspecies of the protozoan parasite *Trypanosoma brucei* (*T.b.*) and transmitted through tsetse flies (*Glossina sp.*). *T.b. gambiense* occurs from West to Central sub-Sahara Africa, while *T.b. rhodesiense* is endemic in East sub-Sahara Africa. Other closely related taxa (*T.b. brucei*, *T. evansi*, *T. equiperdum*, *T. congolense*, *T. vivax*) cause animal African trypanosomiases in domestic animals like cattle, horse, camel, goat, sheep, and buffalo in Africa, Latin America, Europe, and Asia [1].

Approximately 10,000 sleeping sickness patients are diagnosed and treated each year [2]. This is only a fraction of those carrying this lethal infection since (i) the population at risk lives mainly in remote areas outside the action radius of health centres or mobile teams, and (ii) the diagnostic tests suffer from limited sensitivity [3],[4].

There is a consensus to (i) only treat patients with confirmed diagnosis, i.e., in whom the parasite has been demonstrated, except in particular situations, and (ii) to examine the cerebrospinal fluid (CSF) for white blood cell counts and the presence of trypanosomes in order to decide which drugs to administer. To determine whether a patient is cured, examination of CSF is repeated each semester for up to 2 years [4].

In *T.b. gambiense* sleeping sickness, general low parasite loads necessitate the use of concentration techniques to reveal infection. Capillary tube centrifugation (CTC, WOO) [5], quantitative buffy coat (QBC) [6], and mini anion exchange centrifugation technique (mAECT) [7] are applied on blood. mAECT is the most sensitive method for trypanosome detection in blood and is based on a purification technique first described by Lanham et al. and later adapted for diagnosis of sleeping sickness and of animal infections with *T. brucei* and *T. evansi* [7]–[10]. In mAECT, trypanosomes are separated from 350 µl of blood by anion exchange chromatography on diethylaminoethyl cellulose (DEAE). Eluted trypanosomes are then concentrated by low speed centrifugation followed by direct microscopic examination of the sediment in a transparent collector tube. The large volume of blood examined allows detection of fewer than 50 trypanosomes/ml. For sensitive detection of trypanosomes in CSF, single and double centrifugation (DC) and modified single centrifugation (MSC) are applied, with MSC being easier to perform and at least as sensitive as DC [11].

For several years, mAECT was produced at the Projet de Recherches Cliniques sur la Trypanosomiase (PRCT) in Daloa, Côte d'Ivoire, with financial support from the World Health Organization (WHO), and then for some time at Institut Pierre Richet (IPR) in Bouaké, Côte d'Ivoire. The PRCT model is still produced on demand in this country (B. Miezan, personal commmunication). Several attempts to establish a production unit elsewhere were discontinued.

At the request of the Belgian Technical Cooperation (BTC) for the Programme National de Lutte contre la Trypanosomiase Humaine Africaine (PNLTHA) in the Democratic Republic of the Congo (DRC), the Institute of Tropical Medicine (ITM, Belgium) and the Institut National de Recherche Biomédicale (INRB, DRC) developed a modified version of the mAECT. With financial support from the Belgian Directorate General for Development Cooperation and WHO, production at INRB started in 2003. At the end of 2005, production stopped since raw material for the 9-mm diameter glass collector tube was not commercially available anymore.

From 2006 on, with support from the Foundation for Innovative New Diagnostics (FIND), the column rack, microscope viewing chamber, and collector tube were redesigned. Important advantages of the new collector tube and the viewing chamber are their robustness and the fact that microscopic examination of the sediment is possible without mounting the tip of the collector tube under water (Figures 1–3). Improvements were made to the packing trays (Figure S1), filter material (Figure S2), sterilisation procedure, and instruction leaflets. In addition, the production infrastructure at INRB and the quality control system were upgraded. Actually, quality control is performed internally at INRB and externally at ITM before a batch is released. Detailed production standard operating procedures were written and are collated in an mAECT handbook that can be found on the FIND Web site [12].

**Figure 1:**
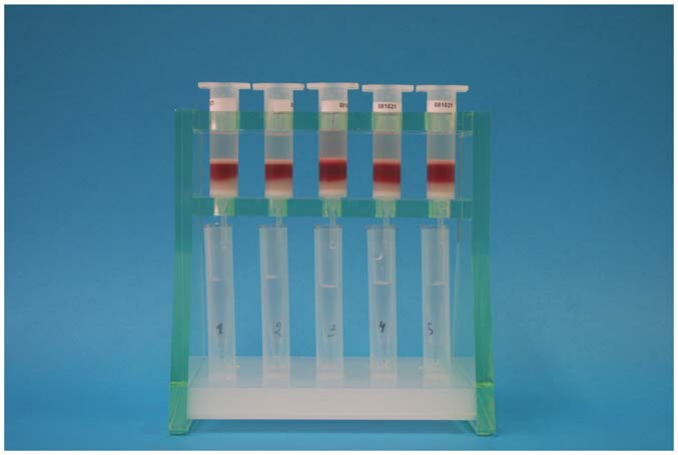
Column rack with mounted columns and trypanosomes being eluted from the blood in the collector tubes.

**Figure 2 pntd-0000471-g002:**
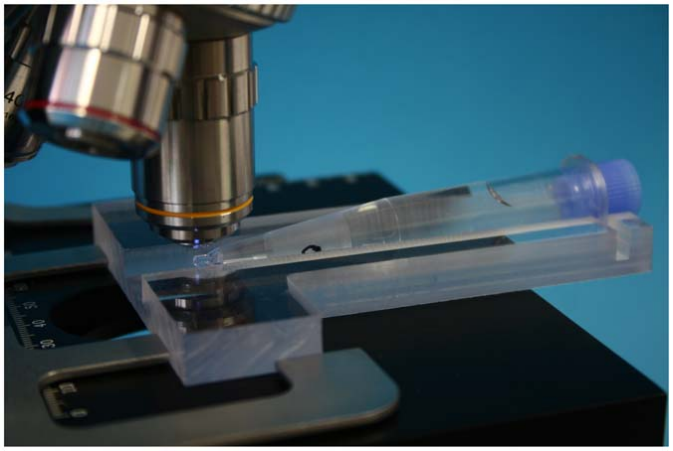
Collector tube mounted in the mAECT viewing chamber under the microscope.

**Figure 3:**
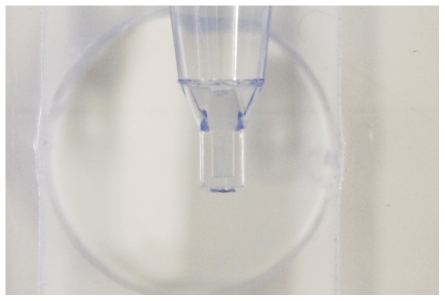
Detail of collector tube tip.

In 2007, a comparison between the first model of mAECT produced at INRB and the PRCT model was carried out at the Centre International de Recherche-Développement sur l'Elevage en zone Sub-humide (CIRDES) in Bobo-Dioulasso, Burkina Faso, on 198 columns of each model. Columns received 350 µl of human blood containing on average 35 *T.b. gambiense* trypanosomes (100 trypanosomes per ml of blood). The time to run a column was recorded and trypanosomes in each collector tube were counted by two independent observers. Averages were compared using paired *t*-test and showed a significantly shorter run time for the INRB than the PRCT model (29 and 42 min, respectively, *p*<0.0001), which is explained by the fact that in the INRB model, no glucose should be added before applying the blood. In addition, more trypanosomes were recovered by the INRB than by the PRCT model (5.85 and 4.92, respectively, *p*<0.005). After changing the filter material in 2008, the second INRB model was compared with the previous one by the same CIRDES experts on 195 columns of each type. The second model runs faster than the first (21 and 33 min, respectively, *p*<0.0001) and showed higher average trypanosome numbers (4.43 and 1.76, respectively, *p*<0.0001). The lower average trypanosome numbers counted with the first model compared to the previous comparison is probably caused by the still unexplained fact that the number of recovered trypanosomes seems to be blood donor dependent.

The INRB mAECT kit consists of two cardboard boxes. One contains 20 mAECT columns best stored at 4°C–8°C, although tests are stable for 12 months at a maximum of 37°C. The other contains 20 collector tubes, 20 centrifugation tubes, 20 transfer pipettes, and the instruction leaflet (available in English, French, and Portuguese). Specifications of the kit are given in Box 1.

**Box 1. Three Advantages and Three Disadvantages for the New mAECT Model**

Advantages

1. Analytical sensitivity <50 trypanosomes per ml of blood at only €3 per test.
2. Robustness and no need to mount collector tube in water for microscopic examination.
3. Applicable in field conditions.

Disadvantages

1. Need for specific material and centrifuge.
2. Limited stability since the glucose is now incorporated in the column buffer (maximum 1 year at 37°C).
3. Qualified personnel needed to perform the test.

In the original MSC test on CSF described by Miezan et al., 2-ml glass pasteur pipettes are used as collector tubes and are examined after mounting the tip under water [11]. With its 4 ml of volume and no need for mounting under water, the new plastic mAECT collector tube is expected to increase the sensitivity and the robustness of MSC on CSF, although a formal comparative test still has to be performed. An MSC kit for 20 tests with instruction leaflets in English, French, or Portuguese is now also available from INRB. Specifications of the kit are given in Box 2.

**Box 2. Three Advantages and Three Disadvantages for the New MSC Model**

Advantages

1. Analytical sensitivity <2 trypanosomes per ml of CSF at only €0.5 per test.
2. Short test time (<15 minutes).
3. Few manipulations.

Disadvantages

1. Need for specific material and centrifuge.
2. CSF sediment cannot be recuperated for other tests.
3. Interference of CSF white blood cells possible, especially at high cell counts.

mAECT and MSC kits can be ordered at INRB. For prices and delivery conditions, contact mumbadieudonne@yahoo.fr. Additional information is available on the FIND Web site at http://www.finddiagnostics.org.

The mAECT and MSC tests are used by diverse organisations and institutes in their HAT control activities and in clinical investigations. Current clients are the National HAT Control Programme in DRC, the World Health Organization, Institute of Tropical Medicine Antwerp, Swiss Tropical Institute, Drugs for Neglected Diseases Initiative, Médecins Sans Frontières, Medische Missie Samenwerking, Institut de Recherche au Développement, and Organisation de Coordination pour la Lutte contre les Endemies en Afrique Centrale.

## Supporting Information

Figure S1 Box with Ten mAECT Columns
(0.26 MB PDF)

Figure S2 Elution of *T. brucei* Parasites from Blood with the mAECT Columns
(0.27 MB PDF)
