# Supplementary figures and images for: Improved Models of Mini Anion Exchange Centrifugation Technique (mAECT) and Modified Single Centrifugation (MSC) for Sleeping Sickness Diagnosis and Staging
Source: PLoS Negl Trop Dis. 2009 Nov 24;3(11):e471. doi: 10.1371/journal.pntd.0000471 (PMC2775158; doi:10.1371/journal.pntd.0000471)

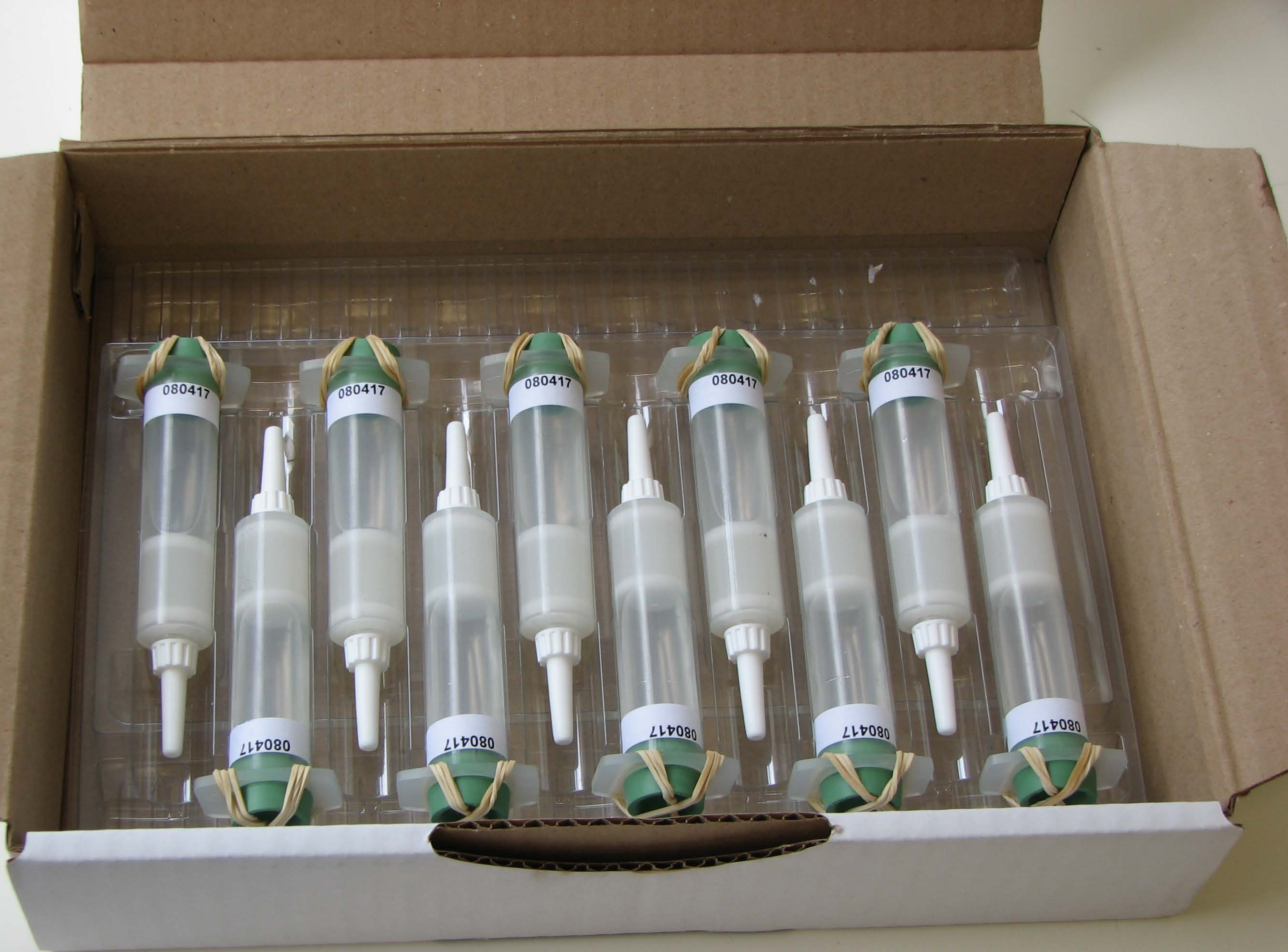

Supplement: Figure S1 — Box with Ten mAECT Columns (0.26 MB PDF) [file pntd.0000471.s001.pdf]

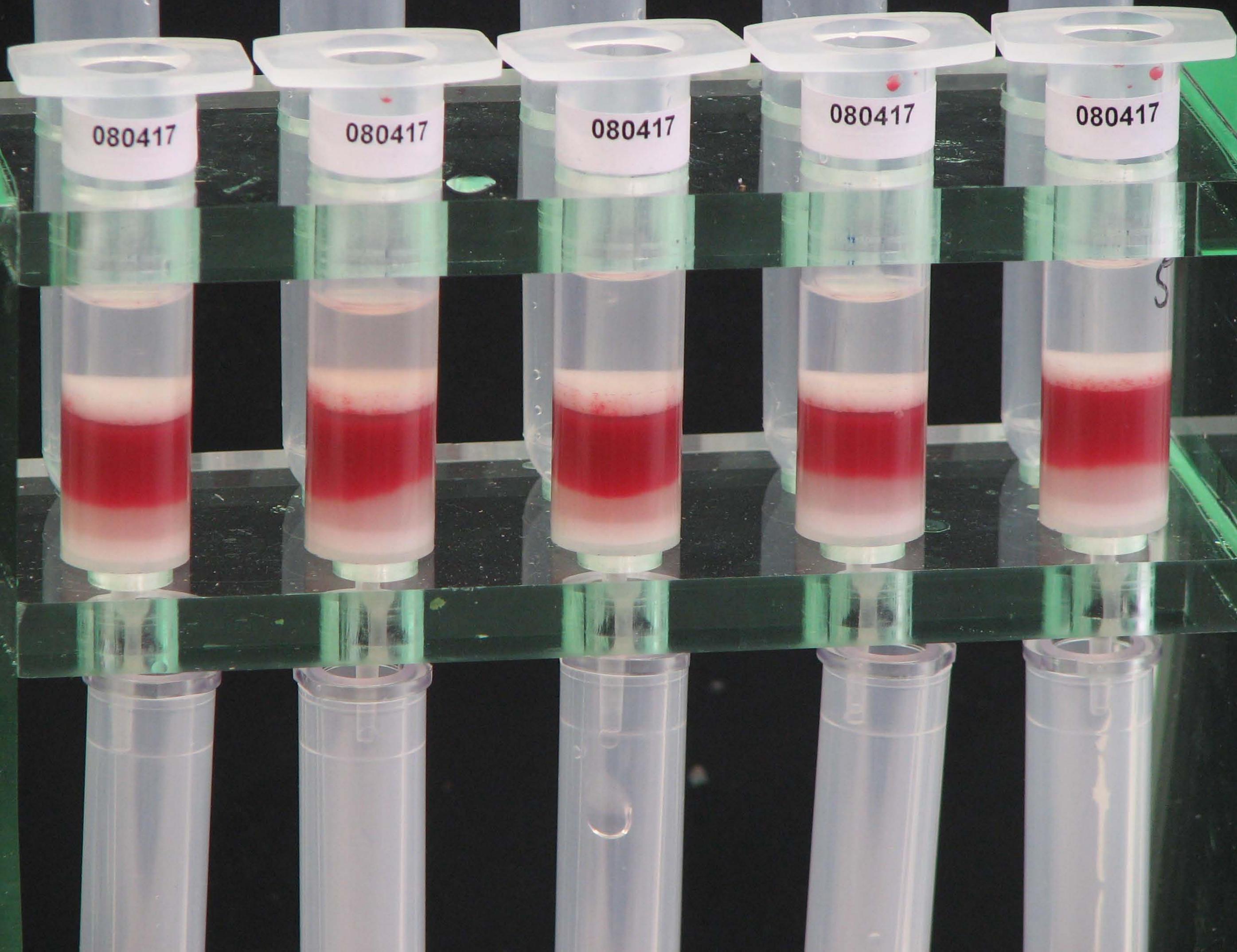

Supplement: Figure S2 — Elution of T. brucei Parasites from Blood with the mAECT Columns (0.27 MB PDF) [file pntd.0000471.s002.pdf]
